# Hypervirulent *Klebsiella pneumoniae* Infections: A Systematic Review of Clinical, Microbiological, and Outcome Data From Reported Cases

**DOI:** 10.1002/mbo3.70352

**Published:** 2026-06-23

**Authors:** Nermin Sakru, Ender Cetinkaya, Feza Irem Aldi, Mervenur Inan, Canan Eryildiz

**Affiliations:** ^1^ Department of Medical Microbiology Trakya University School of Medicine Edirne Türkiye

**Keywords:** bloodstream infection, hvKp, hypervirulent *Klebsiella pneumoniae*, invasive infection, multi‐organ involvement, virulence factors

## Abstract

Hypervirulent *Klebsiella pneumoniae* (hvKp) has been increasingly reported in association with invasive infections and multi‐organ involvement. This systematic review followed PRISMA guidelines and was registered in PROSPERO (CRD420251060200). PubMed, Scopus, and Web of Science were searched for articles published between 2000 and 2025. We conducted a review of published reports to summarize the clinical, microbiological, and outcome characteristics of reported hvKp infections. Studies reporting individual‐level data were included, and descriptive analyses were performed. A total of 778 cases from 376 reports were included. Bloodstream infection and liver involvement were among the most frequently reported clinical features, and multi‐organ involvement was commonly described. Virulence factors, including siderophores and capsular types, were frequently reported; however, microbiological findings varied across studies. Among cases with available data, 82.1% had origin from or travel to endemic regions and, 43.1% had diabetes mellitus. This systematic review provides a descriptive overview of reported hvKp infections, highlighting frequently reported clinical and microbiological patterns. However, the findings are based on heterogeneous and predominantly severe cases and should be interpreted with caution. Further studies using standardized definitions and systematically collected data are needed to better characterize hvKp infections.

## Graphical abstract

Distribution of organ involvement in reported hypervirulent *Klebsiella pneumoniae* cases. Studies restricted to isolated liver abscess cases were excluded. Percentages represent the proportion of cases with documented involvement of each anatomical site among cases with available data. The figure was created using BioRender (https://app.biorender.com) (free). Note: As multiple organ involvements may occur simultaneously in a single patient, individual cases may be counted in more than one category. Therefore, the cumulative frequencies exceed the total number of cases. Percentages were calculated based on cases with available data for each variable.

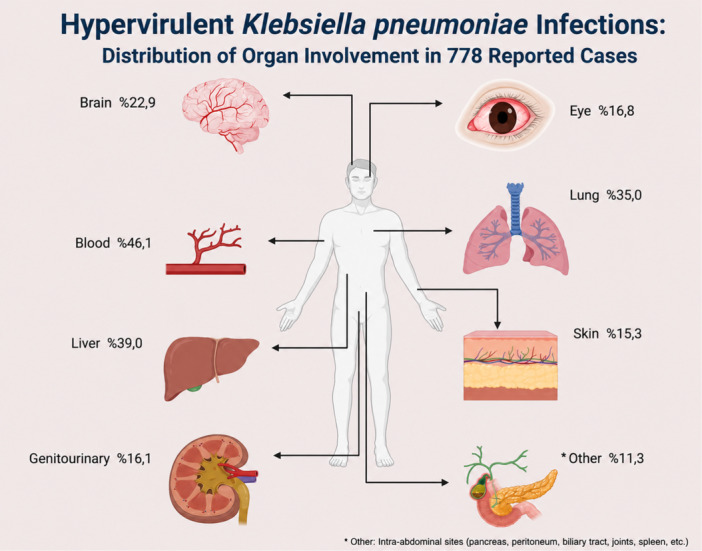

## 1 Introduction

Hypervirulent *Klebsiella pneumoniae* (hvKp), first reported in the Asia‐Pacific region as a cause of community‐acquired invasive infections with metastatic spread, has increasingly been reported worldwide, including in North America and Europe (Moore et al. 2013; Siu et al. 2012). Its overall prevalence among *K. pneumoniae* isolates is 15.2%, exceeding 40% in some Asian countries, with travel or migration from endemic regions remaining an important epidemiological indicator (Li et al. 2014).

A standardized definition of hvKp has not yet been established. Early classifications relied on the hypermucoviscous phenotype detected by the string test, but this was later shown to be insufficient, as hypermucoviscosity can occur in classical strains and does not include all hvKp isolates (Fang et al. 2004; Catalán‐Nájera et al. 2017). Current consensus supports a multidimensional definition integrating phenotypic, genotypic, and clinical features; however, the lack of an internationally standardized definition continues to hinder epidemiological comparability (Russo et al. 2018; Chen et al. 2023a).

Hypervirulent *K. pneumoniae* causes a broad clinical spectrum, marked by metastatic spread and multiple abscess formation. While pyogenic liver abscess is the most common manifestation, hvKp can also lead to pneumonia, necrotizing fasciitis, endophthalmitis, and meningitis (Russo and Marr 2019). Compared with classical strains, it exhibits increased capsule production, enhanced siderophore synthesis, and resistance to phagocytosis, facilitating immune evasion (Hetta et al. 2025).

Despite their generally susceptible profile, early identification of hypervirulent isolates is crucial for effective therapy and source control (Garcia‐Cobos et al. 2025). The hypermucoviscous phenotype is commonly associated with hvKp and is routinely evaluated by the string test (Fang et al. 2004). Among the proposed molecular determinants, *iroB, iucA, peg‐344, rmpA*, and *rmpA2* are regarded as the most robust markers of hypervirulence (Russo et al. 2018).

With its rising incidence, global dissemination, and emerging co‐resistance to critical antibiotics, hvKp represents a growing concern within Gram‐negative infections. This review synthesizes evidence from 2000 to 2025 on its clinical presentation, microbiological characteristics, risk factors, and disease burden.

## 2 Materials and Methods

### 2.1 Study Design

This systematic review was conducted according to the Preferred Reporting Items for Systematic Reviews and Meta‐Analysis (PRISMA) guidelines (Page et al. 2021). The study protocol was prospectively registered in the International Prospective Register of Systematic Reviews (PROSPERO; CRD420251060200).

### 2.2 Search Strategy

Search strategies were developed using combinations of controlled vocabulary terms (e.g., MeSH) and free‐text keywords with Boolean operators to retrieve studies reporting cases of hypervirulent *Klebsiella pneumoniae* (hvKp). The literature search was conducted in PubMed, Scopus, and Web of Science (WoS) databases for studies published between 2000 and 2025 (last updated: 01.07.2025). Keywords included, but were not limited to, “hypervirulent Klebsiella pneumoniae,” “hvKp,” “invasive Klebsiella,” and “hypermucoviscous.” No language restrictions were applied. DeepL and ChatGPT were used to translate non‐English articles.

### 2.3 Eligibility Criteria

#### 2.3.1 Inclusion Criteria

The review focused on the operational definitions used to identify hypervirulent *Klebsiella pneumoniae* (hvKp) across studies. Eligible studies were included whether hvKp identification relied on phenotypic markers (string test), genotypic markers (presence of virulence genes), a combination of both approaches, or clinical presentation (e.g., association with liver abscess).

#### 2.3.2 Exclusion Criteria

Studies not related to hvKp, non‐human or experimental studies, studies with insufficient data, commentaries, book chapters, meeting reports, reviews lacking original data, duplicate publications, studies without accessible full texts, and studies restricted to isolated liver abscess cases were excluded.

### 2.4 Study Selection and Data Management

**Figure 1:**
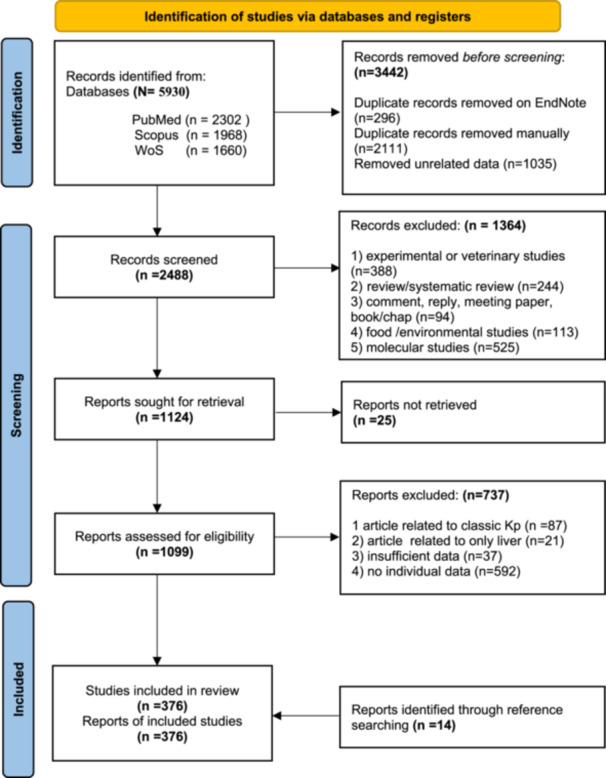
PRISMA 2020 flow diagram for new systematic reviews that included searches of databases and registers only (Page et al. 2021).

All records were exported to EndNote, duplicates removed, and remaining entries transferred to Excel for screening. Reference lists of included articles were manually checked for additional studies. Two independent reviewers assessed full texts for eligibility, resolving discrepancies by consensus. The selection process is summarized in Figure 1.

### 2.5 Data Extraction

Data extraction was performed by one author using a standardized form, and a second author independently cross‐checked all entries. Extracted information included patient demographic characteristics (age, sex, region of origin), medical history, presenting signs and symptoms, diagnostic procedures, therapeutic interventions, clinical outcomes, and follow‐up details.

### 2.6 Quality Assessment

The methodological quality of each study was independently evaluated by two investigators using the Joanna Briggs Institute (JBI) critical appraisal tools.

### 2.7 Statistical Analysis

The extracted data was entered into Microsoft Excel‐files (v2018) and findings were presented as descriptive statistics. Categorical variables were summarized using frequencies and percentages, while continuous variables were reported as mean, standard deviation (SD), median, and interquartile range (IQR).

Detailed information on the search strategy, study selection, data extraction, quality assessment, and the PRISMA abstract checklist is provided in Suporting Information S1.

## 3 Results

### 3.1 Study Characteristics

A total of 5930 records were identified, of which 3442 were excluded before screening due to duplication and/or irrelevance. After screening 2488 records, 1364 were excluded and 25 full texts were inaccessible. Ultimately, 362 studies were included, with an additional 14 identified from reference lists, resulting in 376 reports included in the review (Figure 1, Suporting Information S1: Table S1).

### 3.2 Demographics and Baseline Characteristics

**Figure 2:**
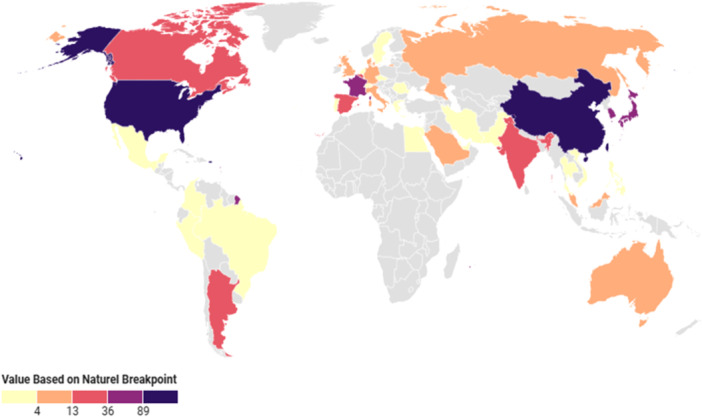
Geographical distribution of reported hypervirulent *Klebsiella pneumoniae* cases by country. The map was created using Datawrapper (https://www.datawrapper.de) (free).

This review included 778 hvKp cases from 40 countries. Most studies originated from China (30.6%), followed by Japan (13.6%) and the United States (13.6%). The majority of cases were reported from China (29.6%), Taiwan (14.5%), and the United States (11.4%), while the remaining 159 studies (42.3%) and 346 cases (44.5%) were from other countries (Figure 2).

The mean age was 55.67 ± 19.07 years (age data missing for 22 cases), and age distribution was similar across anatomical sites. Males accounted for 70.1% of cases, with male predominance observed in all localizations (64.6%–76.1%) (Table 1).

**Table 1:** Epidemiological, microbiological, and diagnostic features.

	Number of cases (%)								
	All Cases (*N *= 778)	Brain (*n* = 178)	Lung (*n* = 272)	Eye (*n* = 131)	Liver(*n* = 303)	Blood (*n* = 359)	Genitourinary (*n* = 125)	Skin (*n* = 119)	Other* (*n* = 88)
Age mean ± SD [No Data]	55.67 ± 19.07 [22]	57.57 ± 19.04 [2]	56.51 ± 17.73 [10]	56.54 ± 13.45	57.19 ± 14.22 [5]	54.13 ± 20.97 [11]	56.34 ± 21.56 [1]	56.83 ± 15.04	56.51 ± 17.67 [3]
Gender [No Data]	[42]	[4]	[20]	—	[3]	[29]	[5]	[2]	[6]
Male	516 (70.1)	124 (71.3)	173 (68.6)	91 (69.5)	224 (74.6)	237 (71.9)	90 (75.0)	89 (76.1)	53 (64.6)
Risk Factors**	**(*N*** = **687)**	**(*n*** = **153)**	**(*n*** = **249)**	**(*n*** = **121)**	**(*n*** = **264)**	**(*n*** = **304)**	**(*n*** = **111)**	**(*n*** = **110)**	**(*n*** = **76)**
Travel history or origin from endemic country	564 (82.1)	129 (84.3)	211 (84.7)	84 (69.4)	198 (75.0)	233 (76.6)	86 (77.5)	90 (81.8)	67 (88.2)
Diabetes mellitus	296 (43.1)	80 (52.3)	97 (39.0)	66 (54.5)	142 (53.8)	134 (44.1)	49 (44.1)	68 (61.8)	27 (35.5)
Hypertension	126 (18.3)	28 (18.3)	43 (17.3)	24 (19.8)	56 (21.2)	48 (15.8)	16 (14.4)	21 (19.1)	15 (19.7)
Immunodeficiency	16 (2.3)	4 (2.6)	5 (2.0)	2 (1.7)	6 (2.3)	8 (2.6)	2 (1.8)	1 (0.9)	4 (5.3)
Malignancy	51 (7.4)	10 (6.5)	14 (5.6)	5 (4.1)	13 (4.9)	28 (9.2)	7 (6.3)	7 (6.4)	6 (7.9)
Alcohol	30 (4.4)	11 (7.2)	9 (3.6)	5 (4.1)	9 (3.4)	8 (2.6)	9 (8.1)	7 (6.4)	1 (1.3)
Others***	112 (16.3)	19 (12.4)	45 (18.1)	8 (6.6)	22 (8.3)	36 (11.8)	25 (22.5)	14 (12.7)	8 (10.5)
Culture Positivity**	**(*N*** = **773/778)**	**(*n*** = **174/178)**	**(*n*** = **271/272)**	**(*n*** = **131/131)**	**(*n*** = **302/303)**	**(*n*** = **356/359)**	**(*n*** = **125/125)**	**(*n*** = **116/119)**	**(*n*** = **88/88)**
Blood	418/462 (90.5)	104/122 (85.2)	153/166 (92.2)	93/104 (89.4)	226/240 (94.2)	343/344 (99.7)	72/83 (86.7)	78/87 (89.7)	45/47 (95.7)
Abscess/Aspirate	301/319 (94.4)	67/73 (91.8)	95/99 (96.0)	102/104 (98.1)	204/209 (97.6)	146/153 (95.4)	48/49 (98.0)	67/73 (91.8)	62/65 (95.4)
Sputum/Bal	153/157 (97.5)	19/21 (90.5)	151/153 (98.7)	16/17 (94.1)	37/37 (100.0)	59/62 (95.2)	16/18 (88.9)	9/9 (100.0)	11/11 (100.0)
Urine	82/100 (82.0)	17/22 (77.3)	31/36 (86.1)	15/20 (75.0)	24/32 (75.0)	52/60 (86.7)	59/69 (85.5)	13/15 (86.7)	6/7 (85.7)
CSF	87/96 (90.6)	92/97 (94.8)	14/20 (70.0)	13/15 (86.7)	44/49 (89.8)	37/42 (88.1)	9/13 (69.2)	5/5 (100.0)	5/6 (83.3)
Tissue/Wound/Swab	55/60 (91.7)	8/12 (66.7)	13/15 (86.7)	14/15 (93.3)	21/22 (95.5)	27/31 (87.1)	15/15 (100.0)	32/35 (91.4)	5/5 (100.0)
Stool	21/23 (91.3)	4/4 (100.0)	13/13 (100.0)	—	3/3 (100.0)	9/10 (90.0)	7/7 (100.0)	4/5 (80.0)	3/3 (100.0)
String Test Positivity**	**(*N*** = **434/484)**	**(*n*** = **70/83)**	**(*n*** = **169/178)**	**(*n*** = **52/55)**	**(*n*** = **158/166)**	**(*n*** = **211/233)**	**(*n*** = **59/68)**	**(*n*** = **72/75)**	**(*n*** = **47/52)**
Capsular Type**	**(*N*** = **469)**	**(*n*** = **45)**	**(*n*** = **165)**	**(*n*** = **56)**	**(*n*** = **158)**	**(*n*** = **207)**	**(n** = **82)**	**(n** = **69)**	**(n** = **48)**
K1	180 (38.4)	14 (31.1)	52 (31.5)	42 (75.0)	106 (67.1)	99 (47.8)	38 (46.3)	25 (36.2)	22 (45.8)
K2	128 (27.3)	17 (37.8)	40 (24.2)	11 (19.6)	37 (23.4)	54 (26.1)	24 (29.3)	22 (31.9)	11 (22.9)
Others****	161 (34.3)	14 (31.1)	73 (44.2)	3 (5.4)	14 (8.9)	54 (26.1)	20 (24.4)	22 (31.9)	15 (31.2)
Sequence Type**	**(*N*** = **401)**	**(*n*** = **40)**	**(*n*** = **163)**	**(*n*** = **28)**	**(*n*** = **101)**	**(*n*** = **177)**	**(*n*** = **62)**	**(*n*** = **36)**	**(*n*** = **46)**
ST11	106 (26.4)	5 (12.5)	64 (39.3)	1 (3.6)	5 (5.0)	35 (19.8)	17 (27.4)	1 (2.8)	10 (21.7)
ST23	94 (23.4)	11 (27.5)	28 (17.2)	11 (39.3)	50 (49.5)	50 (28.2)	21 (33.9)	12 (33.3)	16 (34.8)
ST65	21 (5.2)	2 (5.0)	8 (4.9)	—	6 (5.9)	14 (7.9)	5 (8.1)	2 (5.6)	4 (8.7)
ST86	29 (7.2)	6 (15.0)	11 (6.7)	3 (10.7)	6 (5.9)	7 (4.0)	5 (8.1)	3 (8.3)	1 (2.2)
Others****	151 (37.7)	16 (40.0)	52 (31.9)	13 (46.4)	34 (33.7)	71 (40.1)	14 (22.6)	18 (50.0)	15 (32.6)
Virulance Factors**	**(*N*** = **534)**	**(*n*** = **51)**	**(*n*** = **195)**	**(*n*** = **42)**	**(*n*** = **146)**	**(*n*** = **247)**	**(n** = **88)**	**(n** = **78)**	**(n** = **70)**
Siderophore	419 (78.5)	39 (76.5)	167 (85.6)	29 (69.0)	108 (74.0)	188 (76.1)	70 (79.5)	59 (75.6)	46 (65.7)
Fimbria	207 (38.8)	13 (25.5)	103 (52.8)	7 (16.7)	38 (26.0)	80 (32.4)	35 (39.8)	20 (25.6)	22 (31.4)
Mucoid phenotype	454 (85.0)	50 (98.0)	167 (85.6)	36 (85.7)	125 (85.6)	210 (85.0)	79 (89.8)	69 (88.5)	47 (67.1)
Plasmids	104 (19.5)	9 (17.6)	28 (14.4)	4 (9.5)	10 (6.8)	46 (18.6)	20 (22.7)	9 (11.5)	5 (7.1)
Others****	157 (29.4)	16 (31.4)	44 (22.6)	18 (42.9)	62 (42.5)	72 (29.1)	31 (35.2)	20 (25.6)	34 (48.6)
WGS Performed**	**(*N*** = **179/208)**	**(*n*** = **15/27)**	**(*n*** = **89/95)**	**(*n*** = **6/9)**	**(*n*** = **26/35)**	**(*n*** = **71/78)**	**(n** = **33/37)**	**(n** = **21/28)**	**(n** = **7/10)**
Imaging Techniques Performed**	**(*N*** = **348)**	**(*n*** = **49)**	**(n** = **126)**	**(*n*** = **91)**	**(*n*** = **200)**	**(*n*** = **179)**	**(n** = **70)**	**(n** = **61)**	**(n** = **41)**
CT	320 (92.0)	45 (91.8)	117 (92.9)	84 (92.3)	189 (94.5)	165 (92.2)	66 (94.3)	52 (85.2)	39 (95.1)
MRI	114 (32.8)	28 (57.1)	21 (16.7)	41 (45.1)	75 (37.5)	60 (33.5)	26 (37.1)	25 (41.0)	6 (14.6)
Plain Radiography	67 (19.3)	6 (12.2)	20 (15.9)	15 (16.5)	31 (15.5)	40 (22.3)	17 (24.3)	12 (19.7)	5 (12.2)
USG	113 (32.5)	12 (24.5)	49 (38.9)	42 (46.2)	76 (38.0)	64 (35.8)	16 (22.9)	21 (34.4)	12 (29.3)

Abbreviations: Bal, Bronchoalveolar lavage; CSF, cerebrospinal fluid; CT, computed tomography; MRI, magnetic resonance imaging; SD, standard deviation; USG, ultrasound sonography.

* Other anatomical sites: Involvement of intra‐abdominal sites, including the pancreas, peritoneum, biliary tract, joints, and spleen.

** Percentages are reported according to available data for each variable. Because individual cases could contribute to more than one category (e.g., multiple clinical features, risk factors, microbiological findings, or diagnostic procedures), cumulative counts may exceed the total number of patients included.

*** Other risk factors: Chronic urogenital disease, chronic cardiopulmonary disease, chronic liver disease, chronic neurological disease, hypothyroidism, adrenal insufficiency, beta‐thalassemia major, chronic pancreatitis, and substance abuse.

**** The full list of capsular types, sequence types and virulence factors encompassed in these groups are detailed in Suporting Information S1: Table S1.

### 3.3 Anatomical Locations

The anatomical distribution of 778 hvKp cases is shown in Table 2. Bloodstream involvement was the most frequently reported site of involvement (46.1%), followed by liver (39.0%) and, lung (35.0%). The most frequent concurrent involvements were liver–blood (7.1%), lung–blood (4.2%), and liver–eye (4.1%). Metastatic dissemination to ≥ 3 organs most commonly included the liver (20.1%), followed by blood (18.3%) and lung (14.7%) (Table 2).

**Table 2:** Anatomical locations of isolation of hypervirulent *Klebsiella pneumoniae*.

ORGAN	Brain	Lung	Eye	Liver	Blood	Genitourinary	Skin	Other*
Brain	**50**	—	—	—	—	—	—	—
Lung	3	**96**	—	—	—	—	—	—
Eye	0	2	**2**	—	—	—	—	—
Liver	24	14	32	**0** *	—	—	—	—
Blood	11	33	4	55	**56**	—	—	—
Genitourinary	5	7	4	3	19	**30**	—	—
Skin	1	2	0	6	26	1	**34**	—
Other**	1	1	0	13	13	0	2	**25**
Involvement of ≥ 3 Organs	83	114	87	156	142	56	47	33
Total	**178**	**272**	**131**	**303**	**359**	**125**	**119**	**88**

* Isolated liver abscess cases were excluded

** Other refers to involvement of intra‐abdominal sites, including the pancreas, peritoneum, biliary tract, joints, and spleen

***To avoid duplication, only one direction of each pairwise anatomical combination is shown; reciprocal cells are intentionally left blank.

### 3.4 Clinical Presentation and Risk Factors

Among all cases, 485 were symptomatic (Table 3). Fever was the most common symptom (63.3%) and predominated across anatomical sites, particularly in liver (76.7%), bloodstream (71.6%), and lung (70.4%) involvement. Cardiopulmonary symptoms followed (46.8%), while pain other than abdominal pain (26.4%), gastrointestinal symptoms (22.5%), and central nervous system (CNS) symptoms (19.4%) were less frequent; CNS symptoms were notably common in brain involvement (56.8%). Ocular symptoms occurred in 15.3% overall but in 67.3% of eye involvement. Musculoskeletal, abdominal, and urogenital symptoms were reported in 14.6%, 12.0%, and 9.1% of cases, respectively (Table 3).

**Table 3:** Distribution of presenting symptoms in hypervirulent *Klebsiella pneumoniae* infections by anatomical site.

Symptom**	All cases (*n* = 485)	Brain (*n* = 132)	Lung (*n* = 179)	Eye (*n* = 104)	Liver (*n* = 215)	Blood (*n* = 229)	Genitourinary (*n* = 92)	Skin (*n* = 81)	Other* (*n* = 41)
Fever	307 (63.3)	86 (65.2)	126 (70.4)	73 (70.2)	165 (76.7)	164 (71.6)	65 (70.7)	50 (61.7)	25 (61.0)
Cardiopulmonary symptoms	227 (46.8)	53 (40.2)	119 (66.5)	29 (27.9)	97 (45.1)	106 (46.3)	29 (31.5)	22 (27.2)	15 (36.6)
Non‐abdominal pain	128 (26.4)	46 (34.8)	42 (23.5)	34 (32.7)	69 (32.1)	64 (27.9)	18 (19.6)	42 (51.9)	8 (19.5)
Gastrointestinal symptoms	109 (22.5)	34 (25.8)	37 (20.7)	23 (22.1)	70 (32.6)	59 (25.8)	16 (17.4)	8 (9.9)	17 (41.5)
CNS symptoms	94 (19.4)	75 (56.8)	27 (15.1)	13 (12.5)	38 (17.7)	26 (11.4)	18 (19.6)	3 (3.7)	4 (9.8)
Ocular symptoms	74 (15.3)	22 (16.7)	29 (16.2)	70 (67.3)	53 (24.7)	25 (10.9)	21 (22.8)	3 (3.7)	1 (2.4)
Abdominal pain	58 (12.0)	9 (6.8)	15 (8.4)	18 (17.3)	46 (21.4)	38 (16.6)	12 (13.0)	4 (4.9)	7 (17.1)
Musculoskeletal symptoms	71 (14.6)	14 (10.6)	22 (12.3)	9 (8.7)	32 (14.9)	31 (13.5)	11 (12.0)	38 (46.9)	3 (7.3)
Urogenital symptoms	44 (9.1)	10 (7.6)	15 (8.4)	7 (6.7)	10 (4.7)	12 (5.2)	35 (38.0)	2 (2.5)	1 (2.4)

Abbreviation: CNS, central nervous system.

* Other anatomical sites: Involvement of intra‐abdominal sites, including the pancreas, peritoneum, biliary tract, joints, and spleen.

** Percentages are calculated based on the number of symptomatic cases with available data for each anatomical localization. Since individual patients may present with multiple symptoms concurrently, cumulative counts and percentages across symptom categories may exceed the total number of symptomatic patients.

Among 687 cases with available data, at least one risk factor was identified. The most frequently reported risk factor was origin from or travel to an endemic area (82.1%), followed by diabetes mellitus (43.1%). Hypertension (18.3%), malignancy (7.4%), alcohol use (4.4%), and immunodeficiency (2.3%) were less commonly reported. Overall, risk factor distribution was similar across anatomical sites, although diabetes mellitus was more frequent in cases with skin involvement (61.8%) (Table 1).

### 3.5 Diagnostic Findings

#### 3.5.1 Microbiological Findings

Hypervirulent *K. pneumoniae* was isolated in 773 of 778 cases (99.4%) (Table 1). Blood cultures were positive in 90.5% overall and 99.7% of bloodstream cases. High positivity was also seen in lung (92.2%), liver (94.2%), and eye (89.4%) samples. Abscess/aspirate cultures were positive in 94.4%, particularly eye (98.1%), liver (97.6%), and genitourinary (98.0%) sites. Lower respiratory tract samples (sputum/BAL) were positive in 97.5% (lung 98.7%, liver/skin 100%). Urine positivity was lower, whereas cerebrospinal fluid (CSF) in brain involvement was 94.8%. Tissue, wound, or swab cultures were positive in 91.7% (100% in skin), and stool cultures, though limited, were highly positive.

The string test was positive in 434 of 484 cases (89.7%), with highest rates in eye (94.5%), liver (95.2%), and skin (96.0%). Bloodstream involvement showed 90.6%, whereas brain (84.3%) and genitourinary (86.8%) involvement had lower positivity. Capsular type data were available for 469 cases. K1 and K2 were most frequent (38.4% and 27.3%), with the remaining 34.3% comprising other types (e.g., K5, K16). K1 predominated in eye (75.0%), liver (67.1%), bloodstream (47.8%), genitourinary (46.3%), and other sites (45.8%). Other capsular types were more frequent in lung (44.2%), and K2 predominated in brain (37.8%). Sequence typing data were available for 401 cases. ST11 (26.4%) and ST23 (23.4%) were most frequent overall, with 37.7% representing other types (e.g., ST14, ST15). ST23 predominated in liver (49.5%), eye (39.3%), genitourinary (33.9%), skin (33.3%), bloodstream (28.2%), and brain (27.5%), while ST11 was predominant in lung (39.3%).

Virulence factor data were reported for 534 cases. The hypermucoviscous phenotype (85.0%) and siderophore presence (78.5%) were most common. Other virulence genes were reported in 29.4%. Siderophore presence was consistently high, particularly in lung (85.6%), and the hypermucoviscous phenotype was especially frequent in brain (98.0%) and genitourinary (89.8%) involvement. Fimbriae were more frequent in lung (52.8%), plasmids in genitourinary (22.7%) and bloodstream (18.6%). Whole‐genome sequencing (WGS) data were available for 208 cases; analysis was performed in 179 (86.1%). WGS rates were higher in lung (93.7%), bloodstream (91.0%), and genitourinary (89.2%) involvement and lower in brain (55.6%) and eye (66.7%) (Table 1).

#### 3.5.2 Imaging Findings

Imaging data were available for 348 cases. CT was most commonly used (92.0%), followed by MRI (32.8%), US (32.5%), and plain radiography (19.3%). MRI was more frequently used for brain (57.1%) and eye (45.1%), and US for liver (38.0%) and eye (46.2%). CT use was consistently high across all sites (Table 1).

### 3.6 Treatment and Outcomes

Treatment data were available for 488 cases (Table 4). Medical therapy combined with surgery or interventional radiology was most common (62.1%), particularly in eye (80.5%), liver (74.4%), skin (74.5%), and bloodstream (67.3%).

**Table 4:** Treatment methods, clinical outcome, follow‐up and recurrence/relapse rates.

	Number of Cases (%)								
	All Cases** (*N* = 778)	Brain (*n* = 178)	Lung(*n* = 272)	Eye (*n* = 131)	Liver (*n* = 303)	Blood (*n* = 359)	Genitourinary (*n* = 125)	Skin (*n* = 119)	Other* (*n* = 88)
Treatment Methods**	**(*N*** = **488)**	**(*n*** = **105)**	**(*n*** = **196)**	**(*n*** = **113)**	**(*n*** = **242)**	**(*n*** = **248)**	**(*n*** = **87)**	**(*n*** = **98)**	**(*n*** = **51)**
Surgery/IR only	16 (3,3)	2 (1,9)	13 (6,6)	—	1 (0,4)	6 (2,4)	1 (1,1)	2 (2,0)	—
Surgery/IR and medical treatment	303 (62,1)	61 (58,1)	104 (53,1)	91 (80,5)	180 (74,4)	167 (67,3)	56 (64,4)	73 (74,5)	30 (58,8)
Medical treatment only	169 (34,6)	42 (40,0)	79 (40,3)	22 (19,5)	61 (25,2)	75 (30,2)	30 (34,5)	23 (23,5)	21 (41,2)
Clinical Outcome**	**(*N*** = **585)**	**(*n*** = **148)**	**(*n*** = **213)**	**(*n*** = **113)**	**(*n*** = **245)**	**(*n*** = **284)**	**(*n*** = **97)**	**(*n*** = **92)**	**(*n*** = **48)**
Complete Recovery	272 (46,5)	58 (39,2)	96 (45,1)	34 (30,1)	130 (53,1)	130 (45,8)	59 (60,8)	54 (58,7)	22 (45,8)
Sequel	113 (19,3)	32 (21,6)	36 (16,9)	75 (66,4)	73 (29,8)	51 (18,0)	20 (20,6)	14 (15,2)	5 (10,4)
Death	200 (34,2)	58 (39,2)	81 (38,0)	4 (3,5)	42 (17,1)	103 (36,3)	18 (18,6)	24 (26,1)	21 (43,8)
Recurrence/Relapse**	**(*N*** = **84)**	**(*n*** = **16)**	**(*n*** = **30)**	**(*n*** = **22)**	**(*n*** = **39)**	**(*n*** = **37)**	**(*n*** = **29)**	**(*n*** = **25)**	**(*n*** = **6)**
Yes	16 (19,0)	1 (6,3)	5 (16,7)	3 (13,6)	6 (15,4)	9 (24,3)	1 (3,4)	7 (28,0)	3 (50,0)
Follow‐up Duration in Months**	**(*N*** = **87)**	**(*n*** = **29)**	**(*n*** = **33)**	**(*n*** = **28)**	**(*n*** = **38)**	**(*n*** = **49)**	**(*n*** = **15)**	**(*n*** = **15)**	**(*n*** = **7)**
Median(IQR)	4 (2–12)	9 (2–13.5)	4 (2–12)	2 (1.5–6)	3 (2–12)	5 (2–12)	9 (2–12)	6 (2–12)	3 (1–12)

Abbreviations: IR; interventional radiology, IQR; interquartile range.

* Other anatomical sites: Involvement of intra‐abdominal sites, including the pancreas, peritoneum, biliary tract, joints, and spleen.

** Percentages are calculated based on cases with available data for each variable. As individual cases may receive more than one treatment modality and may contribute to multiple outcome categories, cumulative counts may exceed the total number of patients.

Clinical outcomes were reported for 585 cases. Complete recovery occurred in 46.5%, sequelae in 19.3%, and mortality was 34.2%. Mortality was higher in other sites (43.8%), brain (39.2%), lung (38.0%), and bloodstream (36.3%), but lower in eye (3.5%). Sequelae were frequent in eye involvement (66.4%).

Among 84 cases with recurrence/relapse data, 19% experienced recurrence, particularly in other sites (50.0%), skin (28.0%), and bloodstream (24.3%). Follow‐up data were reported for 87 cases, with a median of 4 months (IQR: 2–12), ranging from 2 to 9 months across sites (Table 4).

## 4 Discussion

Hypervirulent *K. pneumoniae* was first identified in Taiwan and reported cases remain concentrated in the Asia‐Pacific region, but its intercontinental spread has made it a global public health threat.

Major risk factors include diabetes mellitus, malignancy, travel to endemic areas, and Asian origin (Enciu et al. 2025; Serban et al. 2021). In this review, 82.1% of 778 cases were linked to endemic regions or related travel, consistent with the classical pattern. However, the reporting of 44.5% of cases from countries outside Asia indicates that hvKp cases have been reported globally; however, this distribution may reflect publication patterns and data availability rather than true epidemiology (Alba‐Loureiro et al. 2007).

Clinically, the infection most often presents as a liver abscess, with metastatic spread to the eye, lung, and central nervous system in about one‐third of cases (Choby et al. 2020; Bächler et al. 2016). Less common sites include soft tissue, bone, prostate, and spleen, while rare complications such as vascular thrombosis and endocarditis have been described (Choby et al. 2020; Ren et al. 2020; Li et al. 2023; Zhou et al. 2021). Fever, malaise, and abdominal pain are the predominant symptoms, whereas metastatic involvement leads to site‐specific manifestations such as blurred vision or altered consciousness (Serban et al. 2021; Choby et al. 2020; Russo and Marr 2019). In our analysis, bloodstream involvement was the most frequently reported site of involvement; however, this does not necessarily indicate the primary focus of infection, as multiple sites were often reported per case.

In microbiological diagnosis, culture isolation is essential. Hypervirulent *K. pneumoniae* shows high recovery rates from invasive specimens, with blood culture positivity reported at 40%–60% and aspirate cultures at 65%–90%, varying by region. Isolation from respiratory, CNS, tissue, or ocular samples further indicates metastatic spread (Zhou et al. 2021; Sheng et al. 2022). In our review, blood culture positivity was 90.5%, rising to 99.7% among cases with confirmed bloodstream involvement, underscoring bacteremia as a major presentation. Overall, these findings reflect the characteristics of reported cases and may be influenced by selection bias toward severe presentations. Therefore, diagnostic evaluation should include blood cultures alongside samples from the primary and potential metastatic foci to ensure accurate and timely detection.

In phenotypic diagnosis, string test positivity is an important indicator but is not sufficient for definitive diagnosis. Therefore, phenotypic testing should be supported by molecular methods. Whole‐genome sequencing (WGS) enables molecular confirmation of virulence factors and provides a valuable approach for confirming hypervirulence. Major virulence determinants identified in *K. pneumoniae* include capsule formation, fimbrial adhesion, and siderophore production. Genes associated with these virulence factors include *iucA, iutA, iroB, entB, ybtS, rmpA, kfu, allS, magA, fimA/B/C*, and *mrkD* (Russo and Marr 2019). In this review, string test positivity was reported at a high rate (89.7%), indicating that the hypermucoviscous phenotype is a common feature in hvKp infections. When capsule type distribution was evaluated, K1 (38.4%) and K2 (27.3%) were the most frequently identified types among cases with available capsular data. With respect to virulence characteristics, a hypermucoid phenotype (85.0%) and siderophore presence (78.5%) were frequently reported across anatomical sites. However, differences in detection methods and definitions across studies limit comparability. Taken together, these findings indicate that, in defining hvKp, evaluation of the string test, capsule type, and major virulence factors in combination, rather than individually, provides a more meaningful approach for characterizing hypervirulent isolates. In this context, the fact that capsule type and virulence factors were molecularly assessed in a large proportion of cases with available WGS data (86.1%) reflects the increasingly widespread adoption of genome‐based approaches in hvKp research.

In the global distribution of hypervirulent strains, MLST (Multi‐Locus Sequence Typing) analyses have shown that certain sequence types are epidemiologically dominant. Isolates with the K1 capsule type predominantly belong to the ST23 clone and represent leading causes of community‐acquired invasive infections in many regions, particularly in Asia (Tian et al. 2025; Kamau et al. 2022). In this study, ST23 was more frequently reported in cases with invasive involvement; however, no statistical inference can be made from these descriptive data. ST11 was more commonly reported in cases with lung involvement, based on descriptive frequencies

Imaging modalities play a complementary role in diagnosis. Ultrasonography is a sensitive and accessible first‐line tool; computed tomography is superior in demonstrating abscess morphology, gas formation, and extension to adjacent structures; and magnetic resonance imaging provides higher resolution in complicated cases. In addition, abscess drainage performed under ultrasonographic or CT guidance offers therapeutic benefit (Enciu et al. 2025; Bächler et al. 2016; Ren et al. 2020; Li et al. 2023). In this review, computed tomography was used in 92.0% of cases with available imaging data, consistent with its ability to rapidly demonstrate the anatomical extent of infection. Magnetic resonance imaging was more frequently preferred in cases with central nervous system or orbital involvement, reflecting its capacity to evaluate parenchymal changes and vascular complications in detail. Ultrasonography, which was commonly used in cases with liver involvement, plays an important role in clinical management by contributing both to diagnostic assessment and to guidance of percutaneous drainage procedures. Overall, while computed tomography stands out as the primary modality for initial evaluation, imaging strategies appear to be individualized according to clinical requirements and anatomical localization.

Two main principles guide treatment: source control (percutaneous aspiration or catheter drainage, and surgery in selected cases) and appropriate antimicrobial therapy. Percutaneous drainage has demonstrated additional benefit in abscesses ≥ 5 cm in diameter (Nie et al. 2022). Evaluation of the cases included in this review shows that the predominant treatment approach in hvKp infections was medical therapy combined with surgical or interventional radiology procedures (62.1%). These findings are consistent with current clinical practice, in which antibiotic therapy combined with effective source control is considered an important component of management.

Although mortality has generally decreased in hvKp‐associated liver abscess with timely drainage and appropriate antibiotic therapy, rates of death and sequelae increase markedly in the presence of metastatic dissemination, sepsis, or carbapenem‐resistant hvKp (Choby et al. 2020; Chen et al. 2023b, 2022). Approximately 35% of bloodstream infections caused by hypervirulent isolates have been reported to follow a fatal course (Li et al. 2018). The 34.2% mortality rate identified in this review indicates that these infections carry substantial clinical risk. Among cases with ocular or central nervous system metastasis, permanent damage develops in approximately 70% (Chou and Kou 1996; Fang et al. 2007). In this review, mortality among cases with ocular involvement was low (3.5%); however, the markedly high sequela rate (66.4%) indicates that morbidity rather than mortality predominates in ocular involvement. Recurrence has been associated with inadequate drainage, short treatment duration, diabetes, and vascular complications (Serban et al. 2021; Li et al. 2025). The 19.0% recurrence/relapse rate reported in this review, and the fact that this rate reached higher values in certain anatomical localizations. The higher mortality observed particularly in brain, lung, and bloodstream involvement supports the decisive impact of metastatic and systemic spread on clinical outcomes. Early screening for extrahepatic foci and timely implementation of appropriate drainage strategies significantly reduce mortality risk (Serban et al. 2021; Chen et al. 2023b; Hussain et al. 2020). Considering the median follow‐up duration of 4 months, hvKp infections should not be evaluated solely within the acute phase; longer‐term clinical follow‐up should be planned, particularly in high‐risk involvement patterns.

This review has important limitations. Most included studies were case reports or small series, resulting in heterogeneous and predominantly descriptive data; therefore, no meta‐analysis was performed. Incomplete and non‐standardized reporting limited evaluation of some variables. Publication bias cannot be excluded, and the predominance of Asia‐Pacific cases may restrict global generalizability. The included studies used heterogeneous definitions of hvKp, including phenotypic (e.g., string test), genotypic (virulence genes), and clinical criteria. No stratified analysis according to these definitions was performed, which may have resulted in the inclusion of biologically distinct entities within the same analytical framework. Larger multicenter studies with standardized criteria are needed. The dataset is predominantly composed of case reports and small case series, which are inherently subject to selection bias toward severe and unusual presentations. Therefore, the observed frequencies of bloodstream involvement, multi‐organ disease, and mortality are unlikely to represent true population‐level epidemiology.

## 5 Conclusion

In conclusion, this systematic review provides a descriptive overview of reported hvKp infections worldwide. The findings suggest frequent reporting of bloodstream involvement and multi‐organ disease; however, these observations are derived from heterogeneous and predominantly severe cases and should not be interpreted as representative of the overall epidemiology. Due to the lack of a standardized definition of hvKp and the descriptive nature of the data, no causal or associative inferences can be made. Future studies using standardized diagnostic criteria and systematically collected datasets are needed to better define the clinical and microbiological spectrum of hvKp infections.

## Author Contributions

**Nermin Sakru:** conceptualization, data curation, funding acquisition, investigation, methodology, project administration, resources, supervision; writing – review and editing. **Ender Cetinkaya:** data curation, formal analysis, software, visualization, writing – original draft. **Feza Irem Aldi:** data curation, formal analysis, visualization, writing – review and editing. **Mervenur Inan:** data curation, formal analysis, visualization, writing – original draft. **Canan Eryildiz:** conceptualization, investigation, methodology, supervision, project administration, validation, writing – review and editing.

## Funding

The authors have nothing to report.

## Ethics Statement

The authors have nothing to report.

## Conflicts of Interest

The authors declare no Conflicts of Interest.

## Supporting information

Supporting File

## Data Availability

The data that supports the findings of this study are available in Supporting Information S1. All data generated or analyzed during this study are included in this published article and its Supporting Information S1.
